# Supplementary material for: Phosphodiesterase 5 inhibition improves contractile function and restores transverse tubule loss and catecholamine responsiveness in heart failure
Source: Sci Rep. 2019 May 1;9:6801. doi: 10.1038/s41598-019-42592-1 (PMC6494852; doi:10.1038/s41598-019-42592-1)
Supplement: Supplementary file 1 — Supplemental Information [file 41598_2019_42592_MOESM1_ESM.pdf]

Supplemental Information

Phosphodiesterase 5 inhibition improves contractile function and restores transverse tubule loss and catecholamine responsiveness in heart failure.

Michael Lawless[1], Jessica L. Caldwell[1], Charlotte E.R. Smith[2], Emma J. Radcliffe[2], George W.P. Madders, David C. Hutchings, Lori S. Woods, Stephanie J. Church[3], Richard D. Unwin[3], Graeme J. Kirkwood, Lorenz K. Becker, Charles M. Pearman, Rebecca F. Taylor, David A. Eisner, Katharine M. Dibb & Andrew. W Trafford[*].

[1], contributed equally; [2], contributed equally.

* Correspondence

Unit of Cardiac Physiology and [3.] Centre for Advanced Discovery and Experimental Therapeutics, Division of Cardiovascular Sciences, School of Medical Sciences, Faculty of Biology, Medicine and Health, The University of Manchester, Manchester Academic Health Science Centre, Manchester Academic Health Science Centre, 3.24 Core Technology Facility, 46 Grafton Street, Manchester, M13 9NT,
United Kingdom.

+44 161 275 7969

Andrew.W.Trafford@manchester.ac.uk

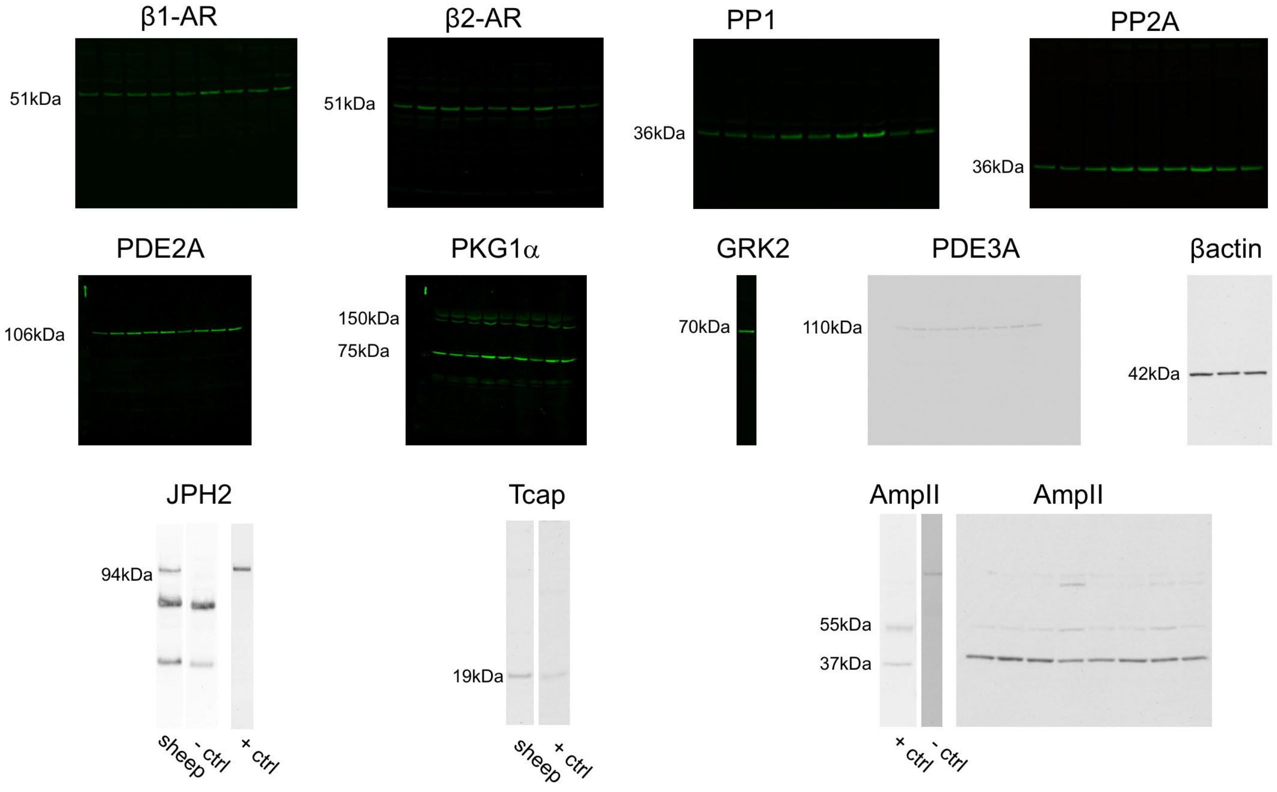

Supplemental Figure 1. Full length blots.

Full length blots of proteins of interest obtained using either a fluorescence or chemiluminescence based image capture system. Proteins and molecular weights as indicated. +ctrl denotes positive control (rat foetal heart for Tcap; mouse liver for MTM1 and rat skeletal muscle for AmpII); - ctrl denotes negative control (secondary antibody only).

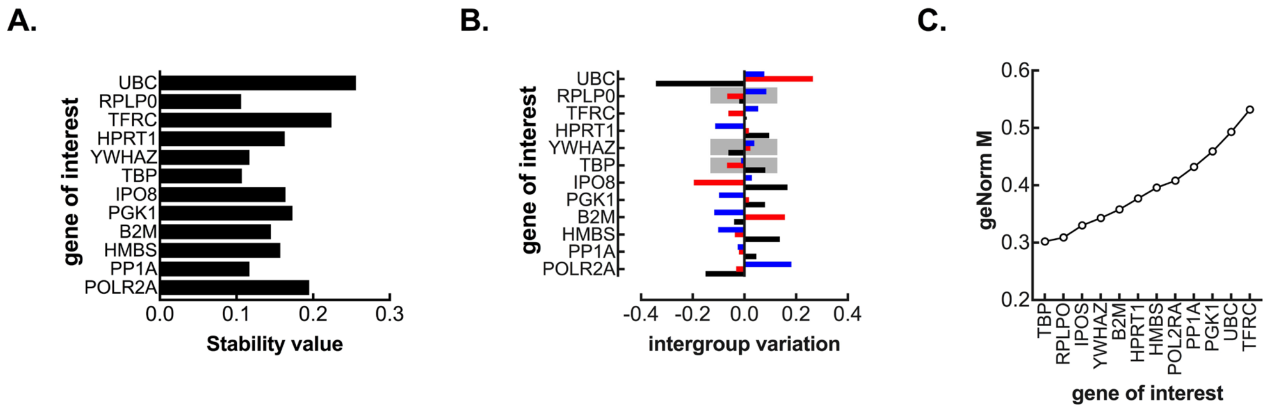

Supplemental Figure 2: Housekeeping genes used for quantitative PCR normalisation in sheep ventricular samples.

A. NormFinder stability values for genes evaluated as potential normalisation controls. **B.** NormFinder intergroup variation values for genes evaluated as potential normalisation controls (black, control; red, heart failure; blue, tadalafil). The highlighted genes were selected as normalisation controls. **C.** geNorm stability values for genes indicated.

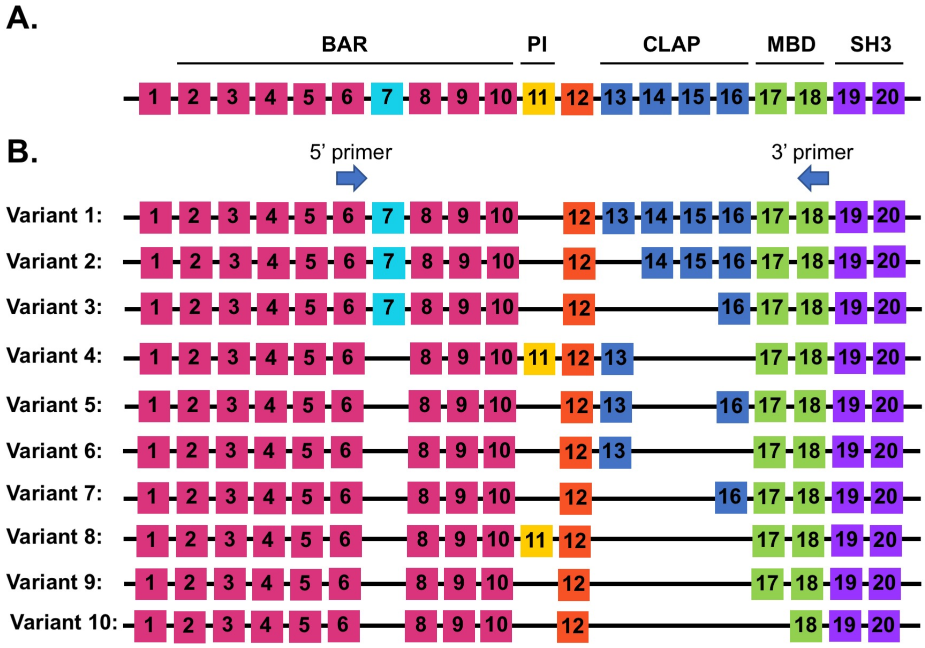

C.

variant	amplicon (bp)
1	1020
2	892
3	762
4	736
5	795
6	691
7	670
8	608
9	563
10	495

Supplemental Figure 3. Primer design strategy for AmpII isoform detection in sheep ventricular myocardium.

A. Exon structure for AmpII gene based on sheep (accession XM_012147686.2) and human (accession NM_139343.2) sequences. BAR, Bin-Amphiphysin-Rvsp domain; PI, phosphoinositide binding domain; CLAP, clathrin-AP2 domain; MBD, myc binding domain; SH3, src homology domain). **B.** PCR primers were designed to span the variable region between exons 6 and 18. **C.** Predicted amplicon sizes for variants 1 – 10 of AmpII.
